# Identification of functional TF-miRNA-hub gene regulatory network associated with ovarian endometriosis

**DOI:** 10.3389/fgene.2022.998417

**Published:** 2022-09-20

**Authors:** Lu Li, Bo Sun, Yingpu Sun

**Affiliations:** ^1^ Center for Reproductive Medicine, The First Affiliated Hospital of Zhengzhou University, Zhengzhou, Henan, China; ^2^ Henan Key Laboratory of Reproduction and Genetics, The First Affiliated Hospital of Zhengzhou University, Zhengzhou, Henan, China

**Keywords:** Endometriosis, microRNA, transcription factor, HIF1A, bioinformatics analysis

## Abstract

Endometriosis (EMs), one of the most common gynecological diseases, seriously affects the health and wellness of women; however, the underlying pathogenesis remains unclear. This study focused on dysregulated genes and their predicted transcription factors (TFs) and miRNAs, which may provide ideas for further mechanistic research. The microarray expression dataset GSE58178, which included six ovarian endometriosis (OE) samples and six control samples, was downloaded from the Gene Expression Omnibus (GEO) to identify differentially expressed genes (DEGs). Gene Ontology (GO) enrichment and Kyoto Encyclopedia of Genes and Genomes (KEGG) pathway analyses were performed to study the cellular and organism-level functions of DEGs. The protein-protein interaction (PPI) network was built and visualized using Cytoscape, and modules and hub genes were explored using various algorithms. Furthermore, we predicted miRNAs and TFs of hub genes using online databases, and constructed the TF-miRNA-hub gene network. There were 124 upregulated genes and 66 downregulated genes in EMs tissues. GO enrichment analysis showed that DEGs were concentrated in reproductive structure development and collagen-containing extracellular matrix, while KEGG pathway analysis showed that glycolysis/gluconeogenesis and central carbon metabolism in cancer require further exploration. Subsequently, HIF1A, LDHA, PGK1, TFRC, and CD9 were identified as hub genes, 22 miRNAs and 34 TFs were predicted to be upstream regulators of hub genes, and these molecules were pooled together. In addition, we found three key feedback loops in the network, MYC-miR-34a-5p-LDHA, YY1-miR-155-5p-HIF1A, and RELA-miR-93-5p-HIF1A, which may be closely related to OE development. Taken together, our study structured a TF-miRNA-hub gene network to decipher the molecular mechanism of OE, which may provide novel insights for clinical diagnosis and treatment.

## Introduction

Endometriosis (EMs), which features the occurrence of endometrial tissue in extra-uterine locations, is a common benign gynecological disorder that presents in 6%–10% of females of reproductive age (Taylor et al., 2021). Women with EMs often experience dysmenorrhea, long-term pelvic pain, and irregular menstruation, while over 50% are asymptomatic when visiting the clinic for infertility (Saunders and Horne, 2021). In addition, a higher prevalence of multi-systemic effects has been observed in this population, and it has been reported that women with EMs are more likely to develop cardiovascular diseases (Okoth et al., 2021). Since the retrograde menstruation theory was proposed in the 1920s, many other theories have emerged to explain the development of EMs, including coelomic metaplasia, metastasis, and altered cellular immunity (Burney and Giudice, 2012; Vercellini et al., 2014; Saunders and Horne, 2021); however, the cause of this disorder remains elusive.

There are three major subtypes of EMs according to the location of the lesion: superficial peritoneal, ovarian, and deep-infiltrating EMs (Chapron et al., 2019). Among them, ovarian endometriosis (OE) is the most common, and clinicians often use laparoscopic ovarian cystectomy as a treatment modality. However, this inevitably reduces the ovarian reserve, which affects patient fertility (Henes et al., 2018). In addition, the high recurrence rate is an intractable problem, and there have been reports of increased incidence of ovarian cancer in these patients (Lu and Gao, 2021). OE induces considerable mental stress and financial burden to many families, as well as social problems. Due to the mystery surrounding the illness, the etiology of OE has not been elucidated to date, early and reliable molecular markers are lacking, and there are no effective treatments, especially for those who want to have children. Many patients experience years of lag between the onset of OE and the presentation of symptoms until diagnosis, which causes unnecessary suffering and consumes significant public health resources (Husby et al., 2003). Therefore, there is an urgent need to uncover the molecular mechanisms of OE and explore effective prediction and therapeutic targets with high sensitivity and specificity for early detection and treatment, thereby improving patients’ quality of life and reducing corresponding expenses.

MicroRNAs (miRNAs) are small non-coding RNA consisting of 19–25 bases that regulate the expression of genes by silencing them (Lu and Rothenberg, 2018). miRNAs are widely involved in crucial physiological processes and are closely related to pathological processes, such as oncogenesis and metastasis (Vishnoi and Rani, 2017; Saliminejad et al., 2019). Recently, researchers in various fields have considered miRNAs as effective biomarkers for the occurrence and development of diseases. Transcription factors (TFs) are a type of protein complex that bind specifically to gene sequences, thereby regulating their transcription; therefore, TFs are considered key participants in gene regulation (Pope and Medzhitov, 2018). Both TFs and miRNAs can influence the expression of genes, and a comprehensive transcriptional regulatory network between them is crucial for understanding physiological processes and disease pathogenesis (Zhang et al., 2015). Ke et al. (2021) constructed a TF-miRNA-mRNA network and identified several genes and negative feedback loops that were highly related to renal ischemia-reperfusion injury. Bo et al. uncovered the underlying mechanism of myasthenia gravis and predicted 21 potential drugs through the feed-forward loop motif-specific subnetwork (Bo et al., 2021); however, there are few systematic studies on the regulatory network in OE.

In the current study, bioinformatics analysis was performed using public datasets, differentially expressed genes (DEGs) and pathways involved in the molecular mechanism of OE were identified. Moreover, we also constructed a TF-miRNA-hub gene regulatory network to identify potential prognostic markers and therapeutic targets of EMs as well as provide new ideas for exploring the molecular mechanism, clinical diagnosis and treatment of OE by revealing key molecules and regulatory mechanisms.

## Materials and methods

### Data collection and processing

By searching the keyword, we collected the dataset GSE58178 based on the GPL6947 platform (Illumina HumanHT-12 V3.0 expression beadchip) from the Gene Expression Omnibus (GEO) database (http://www.ncbi.nlm.nih.gov/geo/), six control samples and six OE samples were involved. Raw data were downloaded and processed in R (R-project.org/).

### Selection of differentially expressed genes

The Limma package was employed to screen DEGs in the expression matrix with |log fold change (FC)| >1 and adjusted *p*-value < 0.05, as the threshold value. The ggplot2 and heatmap packages were used to visualize DEGs: red dots indicate upregulation and blue dots indicate downregulation in the volcano plot; different colors in the heatmap represent the trend of gene expression in different tissues.

### Enrichment analysis of differentially expressed genes

Gene Ontology (GO; http://geneontology.org) is a free open database for gene function analysis and Kyoto Encyclopedia of Genes and Genomes (KEGG; https://www.kegg.jp/) pathway enrichment is a practical resource for studying cellular and organism level functional information. The ClusterProfiler (Yu et al., 2012) package was used for GO and KEGG analyses of DEGs, an adjusted *p* value <0.05 was considered as a statistically meaningful threshold (P was set at 0.05 as the cutoff value for GO cellular component (CC) terms and KEGG pathway analysis).

### Construction of protein-protein interaction network

A PPI network was built to identify potential interactions of DEGs using the STRING online database (https://www.string-db.org/). DEGs were uploaded and the parameters were set as “*Homo sapiens*” with a combined score >0.4. Cytoscape 3.7.1 (https://cytoscape.org/) was utilized to visualize the PPI, functional modules were explored using MCODE, and the ClueGo plug-in was used for KEGG pathway annotation of modules.

### Identification of hub genes

It is found that the degree of a protein in the PPI network is directly related to the importance of its gene, in other words, nodes with a high degree tend to be critical genes (Chin et al., 2014). CytoHubba is a plugin of Cytoscape, which consists of several topological analysis algorithms, among which, we chose Degree, MNC, and MCC to identify molecules in the central of the network that are also the most important genes in the regulation of disease. The top ten genes from the three algorithms were selected, and a Venn diagram (https://bioinformatics.psb.ugent.be/webtools/Venn/) was drawn to identify the intersection as hub genes.

### TF-miRNA-hub gene regulatory network construction

miRWalk2.0 (Dweep and Gretz, 2015) is a comprehensive analysis tool for target gene prediction, miRNA and gene interaction, and target gene enrichment, which includes the results of two target gene prediction databases: TargetScan and miRDBand. MiRTarBase (Huang et al., 2022) is a database that specifically collects miRNA-mRNA targeting relationships, supported by experimental evidence. Online databases were screened to predict miRNAs that target hub genes, and miRNA-gene pairs that existed in the two databases were retained for further analysis. As a database of transcriptional regulatory relationships built from literature mining, TRRUST (https://www.grnpedia.org/trrust/) contains comprehensive TF and target-gene relationships. Transmir (version 2.0; http://www.cuilab.cn/transmir), a free public online tool for investigating the regulation of miRNAs, was used to predict upstream TFs. Consequently, all TFs, miRNAs, and hub genes were uploaded to Cytoscape to structure a comprehensive TF-miRNA-Hub gene regulatory network.

## Results

### Identification of differentially expressed genes in ovarian endometriosis

**FIGURE 1:**
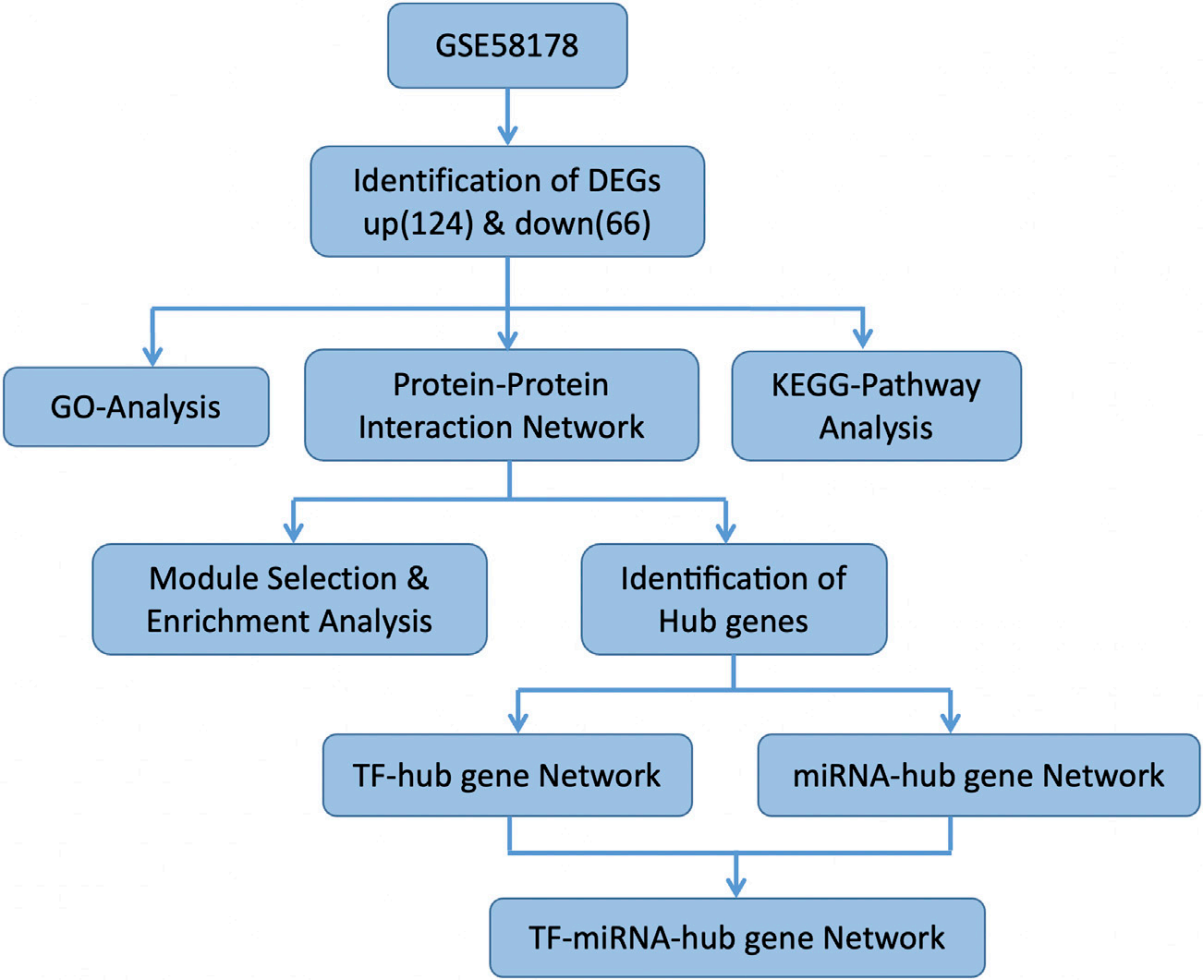
The flow chart of the study.

**FIGURE 2:**
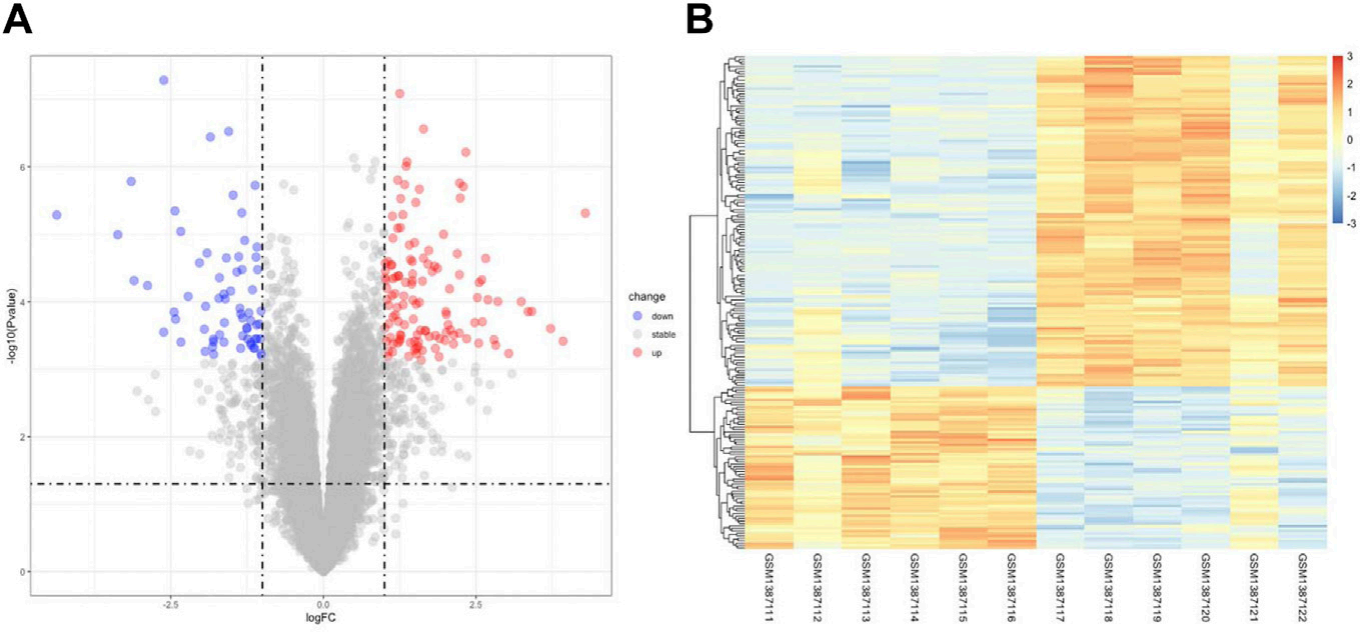
Identification of DEGs Associated with OE. **(A)** the volcano plot of DEGs, red dots indicate upregulated genes and blue dots indicate downregulated genes in OE tissue. **(B)** the heatmap of DEGs, different colors in the heatmap represent the trend of gene expression in different tissues.

Figure 1 illustrates the flow of the study. The dataset GSE58178 was downloaded from GEO and analyzed in R software. There were 190 DEGs between the control and OE samples in total, with 124 upregulated and 66 downregulated, these DEGs may be involved in the pathological processes of EMs (Supplementary Table S1). Volcano plot and heatmap of DEGs are shown in Figures 2A,B, respectively.

### Gene Ontology and Kyoto Encyclopedia of Genes and Genomes enrichment analyses of differentially expressed genes

**FIGURE 3:**
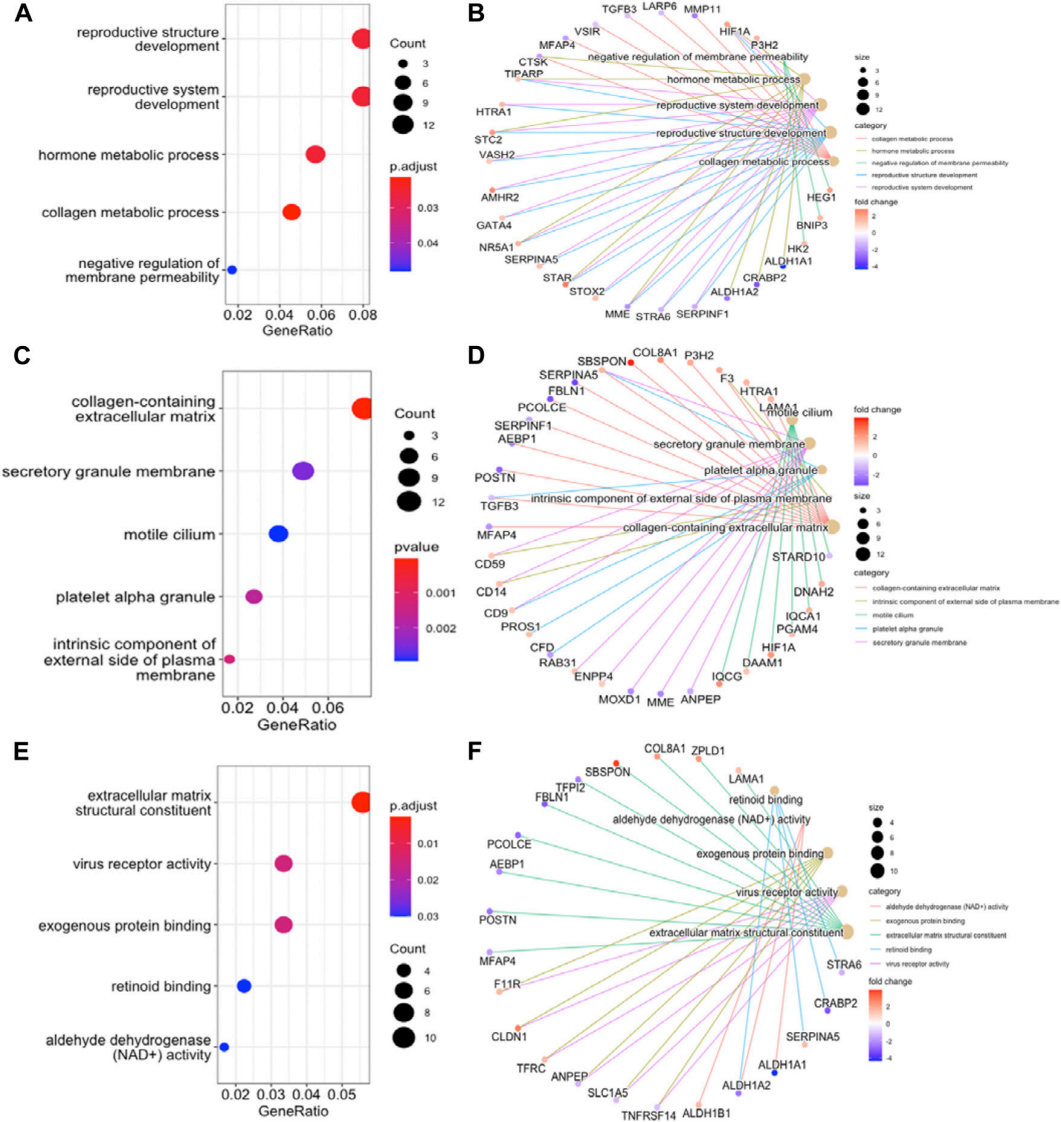
GO Functional Annotation Analysis. The GO analysis displayed top five enriched terms about BP, CC and MF. **(A)**, **(C)**, and **(E)**. dot plot for BP, CC, MF enrichment analysis of DEGs. **(B)**, **(D)**, and **(F)**. the relationship between DEGs and each term. MF, molecular function; BP, biological process; CC, cellular component.

**FIGURE 4:**
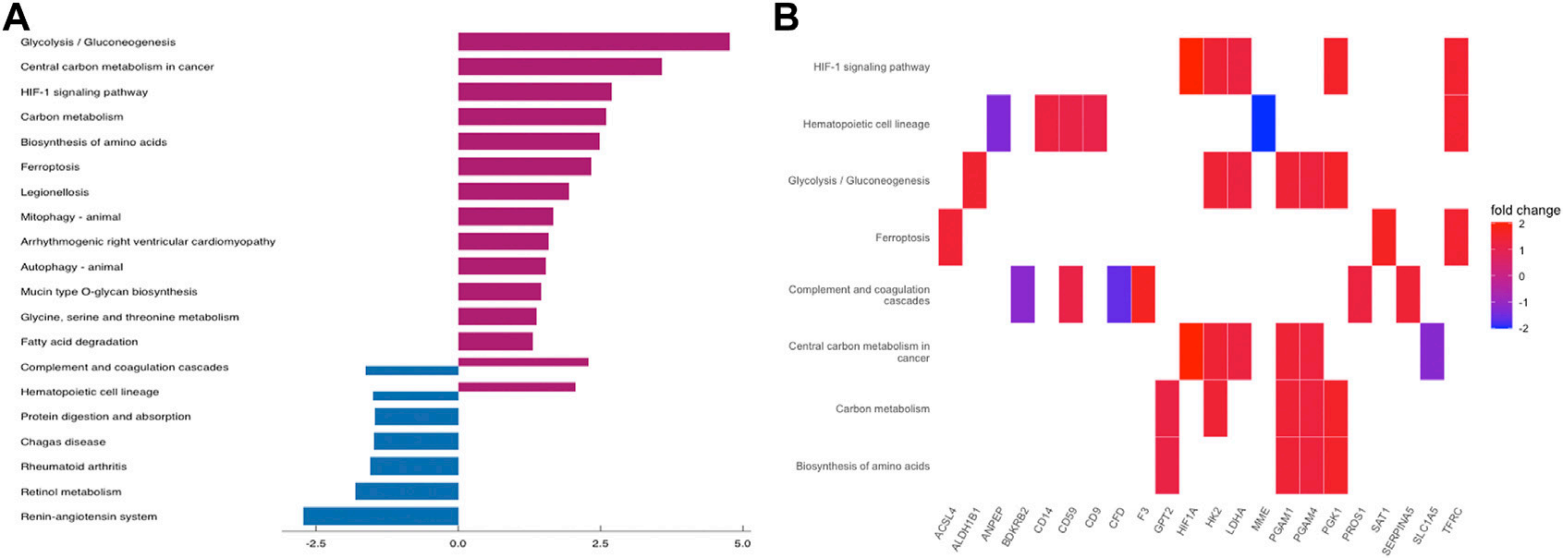
KEGG Pathway Enrichment Analysis. **(A)** bar plot for KEGG pathway enrichment of DEGs. **(B)** the relationship between DEGs and each pathway.

To further understand the function of DEGs, GO and KEGG pathway enrichment analysis were performed to explore the pathways involved and the biological significance related to OE. The biological process (BP) terms were mainly enriched in reproductive structure development, reproductive system development, hormone metabolic processes, and collagen metabolic processes (Figure 3A). The cellular component (CC) terms were mostly involved in collagen−containing extracellular matrix (Figure 3C). Molecular function (MF) analysis demonstrated that the enriched candidates included extracellular matrix structural constituents, virus receptor activity, and exogenous protein binding (Figure 3E). The candidate genes for each term were visualized using the cnetplot package in R (Figures 3B,D,F). The top three KEGG pathways were associated with glycolysis/gluconeogenesis, central carbon metabolism in cancer, and the HIF-1 signaling pathway, and the corresponding candidates in each pathway were also shown using the heatplot package (Figures 4A,B). Taken together, these results imply that DEGs are closely associated with tissue remodeling, migration, and invasion of lesions.

### Protein-protein interaction network construction and module analysis

**FIGURE 5:**
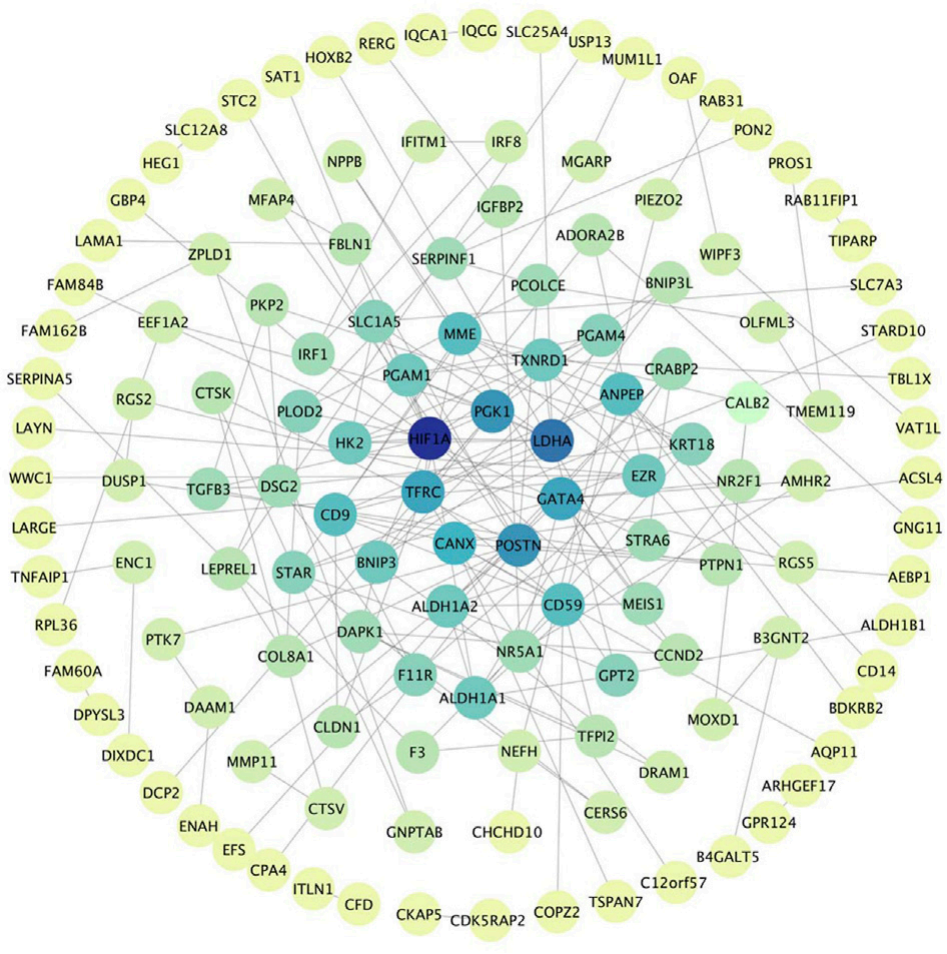
PPI Network, including 133 nodes and 186 edges. The color of a node depends on its degree; the darker the color, the higher the connectivity. Nodes in darker color represent their importance to the network.

**FIGURE 6:**
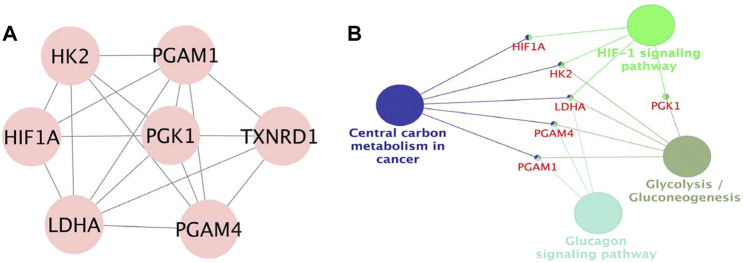
**(A)**. Module in MCODE analysis, the clustering score was 6.0. **(B)**. KEGG pathway analysis of the module.

The PPI network was built by uploading DEGs to STRING, and visualization was performed using Cytoscape. In PPI network, the color of a node depends on its degree; the darker the color, the higher the connectivity, which also indicates the importance of the node in the network (Figure 5). Using the MCODE plugin, we found a key module with seven nodes (HIF1A, HK2, LDHA, PGAM4, PGK1, TXNRD1 and PGAM1) and 18 edges, the clustering score was 6.0 (Figure 6A). KEGG pathway analysis showed that genes in the module were enriched in glycolysis/gluconeogenesis, HIF-1 signaling pathway, central carbon metabolism in cancer, and glucagon signaling pathway (Figure 6B). The PPI network illustrated the interactions between DEGs, and the enrichment results of the key modules were similar to those of the DEGs.

### Hub genes identification and regulatory network construction

**FIGURE 7:**
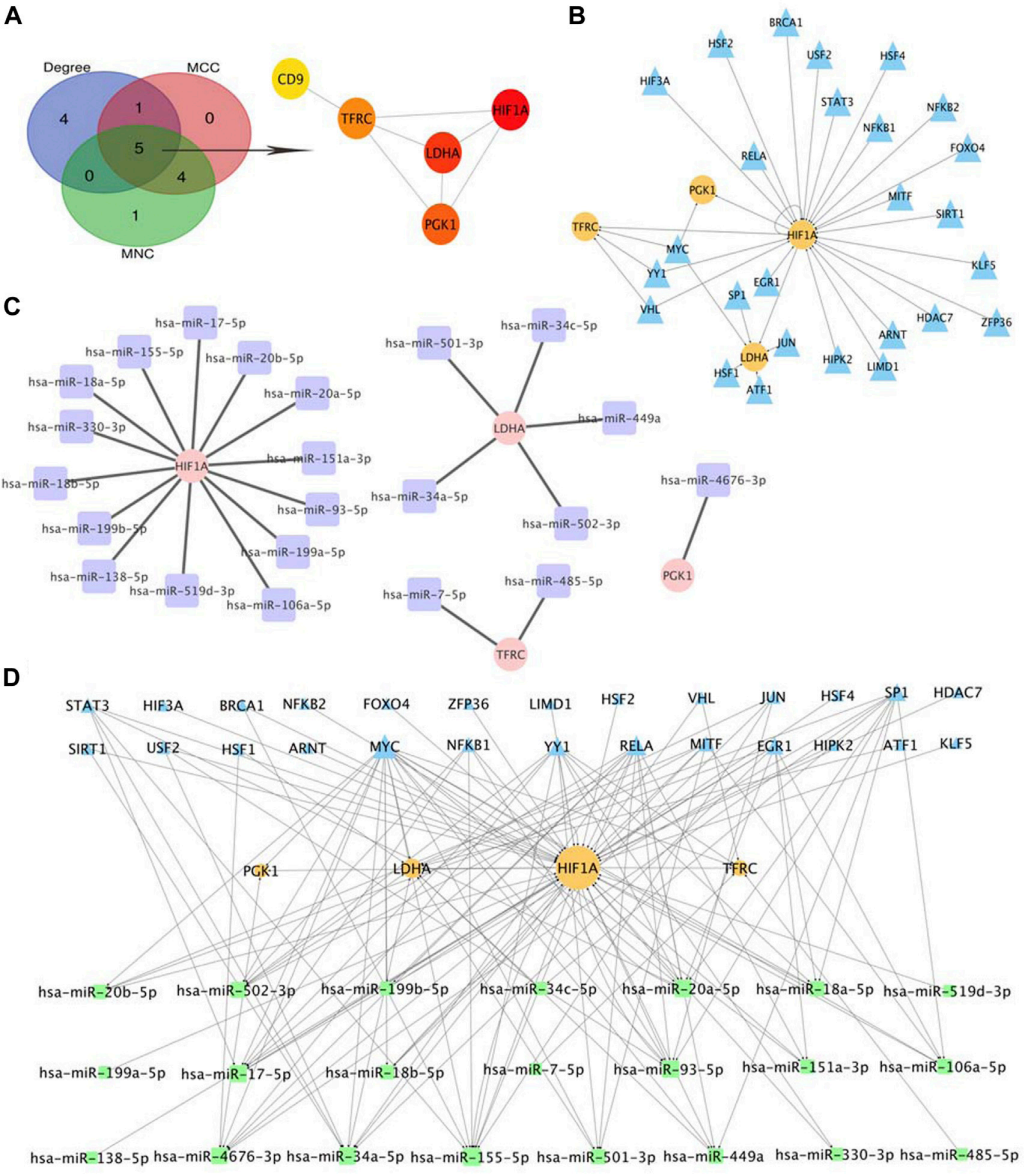
Selection of Hub Genes and Analysis of TF-miRNA-Hub Gene Network. **(A)** hub gene correlated with OE. **(B)** predicted TFs of hub gene, the circles indicate target genes and the triangles indicate predicted TFs. **(C)** predicted miRNAs of hub gene, the circles indicate target genes and the squares indicate predicted miRNAs. **(D)**. Construction of TF-miRNA-hub gene network, the squares indicate predicted miRNAs, the triangles indicate predicted TFs and the circles indicate target genes.

Based on the cytoHubba plugin of Cytoscape, we identified five hub genes in the PPI network: HIF1A, LDHA, TFRC, PGK1, and CD9 (Figure 7A), all of which were upregulated in OE tissues compared with the control. To further decipher the potential molecular mechanisms of EMs, we constructed a TF-miRNA-hub gene network. Through the analysis of the TRRRUST database, a total of 34 upstream TFs were predicted, in which one of the hub genes, HIF1A, was also a TF that could activate the transcription of LDHA, TFRC, and PGK1, and negatively self-regulate (Uchida et al., 2004). Among the identified TFs, MYC regulated PGK1, TFRC, and LDHA, whereas YY1 and VHL were regulators of both TFRC and HIF1A (Figure 7B). Apart from TFs, we also predicted 22 miRNAs that targeted hub genes by searching miRWalk2.0 and miRTarBase, CD9 were excluded from the network because it had no predicted miRNAs that were supported by two databases. HIF1A had the most targeting miRNAs, while PGK1 only had one (Figure 7C). Subsequently, the TF-miRNA-hub gene network was built on the above analysis and was presented in Figure 7D: miR-155-5p had the highest node degree of interaction, with one target and eight TFs; followed by miR-17-5p, miR-20-5p, miR-34a-5p, and miR-93-5p, with one target and seven TFs. Moreover, we also found three regulatory axes, MYC-miR-34a-5p-LDHA, YY1-miR-155-5p-HIF1A, and RELA-miR-93-5p-HIF1A, which may be involved in OE development. These genes, TFs, and miRNAs interact with each other to mediate OE progression. Detailed information on TFs and miRNAs in the network is listed in Supplementary Table S2.

## Discussion

As an estrogen-dependent inflammatory gynecological disease, OE has a variety of adverse effects on women, and for infertile patients seeking assisted reproductive technology (ART) therapy, it may also affect egg quality and embryo implantation, and lead to miscarriage (Harb et al., 2013; Sanchez et al., 2017), which creates a great economic and psychological burden on the family. Due to the high risk of recurrence and carcinoma development (Kajiyama et al., 2019; Wattanayingcharoenchai et al., 2021), it is essential to unravel the specific genes and their regulatory molecules, as well as functional pathways that mediate the changes in their biological processes, thereby potentially improving the clinical management of the disease.

Although tremendous efforts have been made in the past few years to provide new insights into the possible mechanisms of EMs, most studies have concentrated on illuminating the molecular mechanisms associated with protein-coding genes. Recently, miRNAs were considered to play a key role in the biological process of EMs, and TFs were also thought to be closely associated with the development of the disorder (Aznaurova et al., 2014; Nasu et al., 2022), but the regulatory network of these molecules has been less studied in OE. Zhao et al. (2018) established concomitant miRNA-TF-gene regulatory network in their study, 107 differentially expressed miRNA and 6,112 DEGs was screened and included in the next regulatory network analysis. While in our study, instead of put all the DEGs in the network, we used three topological analysis method (degree, MNC, MCC) to identify molecules in the central of the network that are also the most important genes in the regulation of disease, and then analyzed the interactions and regulations between the five hub genes and their upstream miRNAs and TFs. We believe that this analytical process is able to reveal those molecules that really play a crucial role in the regulatory mechanisms of diseases. Consequently, we identified three key motifs that might aid in revealing the underlying pathogenesis of OE, although further experimental validation of these results is needed in the future.

In GO function analysis, the DEGs were involved in “reproductive structure development,” “collagen-containing extracellular matrix, and “extracellular matrix structural constituent,” which explains the properties of EMs adhesion and invasion outside the uterine cavity (Malandrino et al., 2018). In KEGG pathway analysis, the top three pathways were “glycolysis/gluconeogenesis,” “central carbon metabolism in cancer,” and “HIF-1 signaling pathway,” the former two pathways are essential for cellular processes, because they are energy providers (Wong et al., 2017), while the latter pathway is a well-known signaling pathway involved in many physiological and pathological processes in the body and is reportedly associated with EMs (Zhou et al., 2018). Exotic lesions are exposed to a unique peritoneal microenvironment and hypoxic conditions induce steroids and angiogenesis, mediating inflammatory responses and immunosuppression. Abnormally active intracellular glycolysis and increased lactate production are key steps in promoting the occurrence and development of EMs. Their functions should be further investigated as potential therapeutic targets for OE.

Five DEGs in the OE group were identified as hub genes. HIF1A is a major modulator that balances oxygen supply and demand, and can maintain cell survival under hypoxia by inducing angiogenesis and regulating the metabolic adaptation state of cells (Pugh and Ratcliffe, 2003). Recent studies have demonstrated a relationship between hypoxia and EMs, which may increase lesion adhesion, cause inflammatory cytokine production, and suppress the immune response (Li et al., 2021a). Therefore, HIF1A has been recognized as a promising therapeutic target, and several small-molecule compounds have been identified (Ma et al., 2021). LDHA, a rate-limiting enzyme in glycolysis, mediates immune escape by regulating lactate production, promotes cell proliferation, is highly expressed in various cancers, and has been implicated in the progression and prognosis of the OE (Feng et al., 2018). A previous study confirmed that LDHA inhibits apoptosis and promotes migration of endometrial cells; thus, it may be closely related to the development of EMs (Zheng et al., 2021). PGK1 is a key enzyme regulating ATP production in glycolysis and is involved in many signaling pathways as an oncogene, of which the HIFα-PKG1 signaling pathway can mediate epithelial-mesenchymal transition, while the MYC-PGK1 axis accelerates the production of lactate and ATP and promotes cell proliferation and metastasis (He et al., 2019). These genes are critical for the regulation of glycolytic metabolism in EM tissues, and drugs targeting these genes may exert therapeutic effects by lowering cell survival rates, which may be an effective non-hormonal treatment option.

Intracellular iron is also involved in oxygen transport, energy metabolism, and other biological processes. Large amounts of iron promote the proliferation and metastasis of cancer cells (Pu et al., 2022). However, excessive iron concentrations produce excessive reactive oxygen species (ROS), resulting in ferroptosis. Abnormal expression of TFRC, a crucial player in iron metabolism, has been verified in various cancers (Shen et al., 2018) and reported to be over-expressed in peritoneal fluid of women with EM (Li et al., 2021b). Li et al. (2021c) reported that erastin could activate ferroptosis in ectopic endometrial stromal cells (EESC), and then induce EESC death, which may be a novel therapeutic strategy for treating EMs. A recent study showed that ferroptosis may induce VEGF and IL-8 secretion and promote angiogenesis of the lesion (Li et al., 2022). These studies suggest the double-edged sword of ferroptosis in EMs, and how to balance and utilize the important role of iron overload requires further study for the development of targeted therapies.

CD9 belongs to the tetraspanin superfamily. Apart from a variety of biological activities, such as signal transduction, inflammation regulation, and cell adhesion, CD9 is reportedly involved in the oncogenesis and metastasis of cancer (Brosseau et al., 2018). It is also a favorable prognostic predictor and potential therapeutic target (Lorico et al., 2021). A study comparing stromal stem cells (menstrual blood-derived) from females with EMs and non-EMs found higher CD9, CD10, and CD29 expression levels in women with EMs (Nikoo et al., 2014), suggesting that EMs share the same properties as tumor cells, namely the invasive growth of diseased tissue. Similar to the results in our study, the upregulation of the surface marker CD9 could be used as an effective auxiliary detection method for the diagnosis of EMs in the future. Given that there is no effective cure for OE, these small molecules are expected to be potential therapeutic targets for the development of drugs that inhibit the invasive growth of ectopic tissue outside the uterus, thereby treating the disease and alleviating the suffering of patients.

Gene expression is precisely regulated by specific TFs. Herein, we predicted several TFs that may be associated with the pathology of OE. JUN and USF2 are associated with aromatase expression and activity, which have been used as drug targets for the treatment of EMs (Utsunomiya et al., 2008; Yu et al., 2008). SP1 and MYC are well-known cancer regulators, which have been reported to be highly expressed in diseased tissues (Proestling et al., 2015; Shen et al., 2020). NFKB and RELA both belong to the NF-κB signaling pathway, which can activate pro-inflammatory, proliferative, and anti-apoptotic genes, and they have also been confirmed to be related to the development of EMs (Kaponis et al., 2012). The functions of other factors, such as LIMD1, KLF5, HDAC7, MITF, and ZEP36, in OE remain unclear and require further exploration. miRNAs have entered the horizon as potential diagnostic markers for diseases, and circulating miRNAs in plasma and serum are recommended as non-invasive biomarkers for EMs. Many miRNAs have been found to be differentially expressed between EMs and healthy tissues, including miR-138, miR-199-5p, miR-20a, miR-34c, and miR-449 (Bjorkman and Taylor, 2019; Vanhie et al., 2019; Maier and Maier, 2021), which is consistent with our prediction and confirms the reliability of our study. Additionally, our study identified several miRNAs, whose association with EMs has not been previously reported in relevant studies. For instance, miR-502-3p was reported as a tumor suppressor, and its overexpression inhibits the proliferation and differentiation of gallbladder tumor cells (Li et al., 2020). Knockdown of miR-93-5p reportedly suppresses carcinoma of the head and neck and tumor angiogenesis, migration, and invasion (Zhang et al., 2020). Overexpression of miR-485-5p negatively regulates mitochondrial respiration and inhibits the spontaneous metastasis of breast cancer cells *in vivo* (Lou et al., 2016). Therefore, we hypothesized that the abnormal expression of these miRNAs may mediate angiogenesis, cell proliferation, and metastasis of endometriotic lesions, which are part of the epigenetic mechanisms underlying the pathogenesis and development of OE.

miRNAs and TFs share common targets and interact with one another. Consequently, the TF-miRNA-hub gene regulatory network was constructed and three key motifs were identified: MYC-miR-34a-5p-LDHA, YY1-miR-155-5p-HIF1A, and RELA-miR-93-5p-HIF1A. The three miRNAs in the motifs were downregulated in EMs tissues and contributed to the upregulation of HIF1A and LDHA, the functions of which are discussed above (Lv et al., 2015; Nisenblat et al., 2016; Rezk et al., 2021). In addition, previous studies have reported that MYC negatively regulates the expression of miR-34a-5p in multiple myeloma (Xiao et al., 2019), and YY1 levels are inversely related to miR-155 in atherogenesis (Tian et al., 2014). miR-155 may serve as a biomarker in multiple diseases; it is overexpressed in hepatocellular carcinoma (Mohamed et al., 2020) and is closely related to *Helicobacter pylori* infection and the prognosis of gastritis (Oana et al., 2022). Moreover, miR-155 was differentially expressed in patients with EMs, and as a noninvasive predictor of MEs, it is worth exploring in the future. Here, we speculate that these regulators function to explain the underlying pathogenesis of OE: TF negatively regulates the expression of miRNAs, and the reduction of miRNAs results in the loss of repression of downstream target genes, which increases their expression, which in turn activates various pathways, such as the HIF-1 signaling pathway, glycolysis, and ferroptosis, described above. This regulatory series alters the cell metabolism and micro-environment that are closely related to OE occurrence. In summary, these positive-feedback loops may be correlated with pathogenesis and show promise as new targets of OE. However, further experimental studies are required to validate these findings in the future.

There exited some limitations of our study, firstly, we only use a single dataset and the number of sample size was too limited to strengthen the reliability of our results, secondly, no experimental validation was conducted since the OE samples cannot be obtained in our center, we will perform further study once we collected samples from gynecology department in the future.

## Conclusion

Taken together, using bioinformatics analysis, we identified several key genes related to steroid metabolism, hypoxia, and cell growth regulation that might play a crucial role in OE. By building the TF-miRNA-hub gene regulatory network, our research confirmed and significantly extended the findings of previous studies by identifying key miRNAs and TFs associated with OE development, which has important clinical significance for the in-depth understanding of OE, as well as exploring new therapeutic directions.

## Data Availability

Publicly available datasets were analyzed in this study. The names of the repository/repositories and accession number(s) can be found in the article/Supplementary Material.

## References

[B1] Aznaurova Y. B. Zhumataev M. B. Roberts T. K. Aliper A. M. Zhavoronkov A. A. (2014). Molecular aspects of development and regulation of endometriosis. Reprod. Biol. Endocrinol. 12, 50. 10.1186/1477-7827-12-50 24927773 PMC4067518

[B2] Bjorkman S. Taylor H. S. (2019). MicroRNAs in endometriosis: Biological function and emerging biomarker candidates. Biol. Reprod. 100 (5), 1135–1146. 10.1093/biolre/ioz014 30721951 PMC6497525

[B3] Bo C. Zhang H. Cao Y. Lu X. Zhang C. Li S. (2021). Construction of a TF-miRNA-gene feed-forward loop network predicts biomarkers and potential drugs for myasthenia gravis. Sci. Rep. 11 (1), 2416. 10.1038/s41598-021-81962-6 33510225 PMC7843995

[B4] Brosseau C. Colas L. Magnan A. Brouard S. (2018). CD9 tetraspanin: A new pathway for the regulation of inflammation? Front. Immunol. 9, 2316. 10.3389/fimmu.2018.02316 30356731 PMC6189363

[B5] Burney R. O. Giudice L. C. (2012). Pathogenesis and pathophysiology of endometriosis. Fertil. Steril. 98 (3), 511–519. 10.1016/j.fertnstert.2012.06.029 22819144 PMC3836682

[B6] Chapron C. Marcellin L. Borghese B. Santulli P. (2019). Rethinking mechanisms, diagnosis and management of endometriosis. Nat. Rev. Endocrinol. 15 (11), 666–682. 10.1038/s41574-019-0245-z 31488888

[B7] Chin C. H. Chen S. H. Wu H. H. Ho C. W. Ko M. T. Lin C. Y. (2014). cytoHubba: identifying hub objects and sub-networks from complex interactome. BMC Syst. Biol. 8 (4), S11. 10.1186/1752-0509-8-S4-S11 25521941 PMC4290687

[B8] Dweep H. Gretz N. (2015). miRWalk2.0: a comprehensive atlas of microRNA-target interactions. Nat. Methods 12 (8), 697. 10.1038/nmeth.3485 26226356

[B9] Feng Y. Xiong Y. Qiao T. Li X. Jia L. Han Y. (2018). Lactate dehydrogenase A: A key player in carcinogenesis and potential target in cancer therapy. Cancer Med. 7 (12), 6124–6136. 10.1002/cam4.1820 30403008 PMC6308051

[B10] Harb H. M. Gallos I. D. Chu J. Harb M. Coomarasamy A. (2013). The effect of endometriosis on *in vitro* fertilisation outcome: A systematic review and meta-analysis. Bjog 120 (11), 1308–1320. 10.1111/1471-0528.12366 23834505

[B11] He Y. Luo Y. Zhang D. Wang X. Zhang P. Li H. (2019). PGK1-mediated cancer progression and drug resistance. Am. J. Cancer Res. 9 (11), 2280–2302. 31815035 PMC6895440

[B12] Henes M. Engler T. Taran F. A. Brucker S. Rall K. Janz B. (2018). Ovarian cyst removal influences ovarian reserve dependent on histology, size and type of operation. Womens Health (Lond) 14, 1745506518778992. 10.1177/1745506518778992 29806554 PMC5974568

[B13] Huang H. Y. Lin Y. C. Cui S. Huang Y. Tang Y. Xu J. (2022). miRTarBase update 2022: an informative resource for experimentally validated miRNA-target interactions. Nucleic Acids Res. 50 (D1), D222–D230. 10.1093/nar/gkab1079 34850920 PMC8728135

[B14] Husby G. K. Haugen R. S. Moen M. H. (2003). Diagnostic delay in women with pain and endometriosis. Acta Obstet. Gynecol. Scand. 82 (7), 649–653. 10.1034/j.1600-0412.2003.00168.x 12790847

[B15] Kajiyama H. Suzuki S. Yoshihara M. Tamauchi S. Yoshikawa N. Niimi K. (2019). Endometriosis and cancer. Free Radic. Biol. Med. 133, 186–192. 10.1016/j.freeradbiomed.2018.12.015 30562557

[B16] Kaponis A. Iwabe T. Taniguchi F. Ito M. Deura I. Decavalas G. (2012). The role of NF-kappaB in endometriosis. Front. Biosci. 4 (4), 1213–1234. 10.2741/s327 22652867

[B17] Ke P. Qian L. Zhou Y. Feng L. Zhang Z. Zheng C. (2021). Identification of hub genes and transcription factor-miRNA-mRNA pathways in mice and human renal ischemia-reperfusion injury. PeerJ 9, e12375. 10.7717/peerj.12375 34754625 PMC8555504

[B18] Li G. Lin Y. Zhang Y. Gu N. Yang B. Shan S. (2022). Endometrial stromal cell ferroptosis promotes angiogenesis in endometriosis. Cell Death Discov. 8 (1), 29. 10.1038/s41420-022-00821-z 35039492 PMC8763888

[B19] Li S. Zhou Y. Huang Q. Fu X. Zhang L. Gao F. (2021). Iron overload in endometriosis peritoneal fluid induces early embryo ferroptosis mediated by HMOX1. Cell Death Discov. 7 (1), 355. 10.1038/s41420-021-00751-2 34782602 PMC8593044

[B20] Li W. N. Wu M. H. Tsai S. J. (2021). Hypoxia and reproductive health: The role of hypoxia in the development and progression of endometriosis. Reproduction 161 (1), F19–f31. 10.1530/REP-20-0267 33112784

[B21] Li Y. Gong Y. Ma J. Gong X. (2020). Overexpressed circ-RPL15 predicts poor survival and promotes the progression of gastric cancer via regulating miR-502-3p/OLFM4/STAT3 pathway. Biomed. Pharmacother. 127, 110219. 10.1016/j.biopha.2020.110219 32559850

[B22] Li Y. Zeng X. Lu D. Yin M. Shan M. Gao Y. (2021). Erastin induces ferroptosis via ferroportin-mediated iron accumulation in endometriosis. Hum. Reprod. 36 (4), 951–964. 10.1093/humrep/deaa363 33378529

[B23] Lorico A. Lorico-Rappa M. Karbanová J. Corbeil D. Pizzorno G. (2021). CD9, a tetraspanin target for cancer therapy? Exp. Biol. Med. 246 (9), 1121–1138. 10.1177/1535370220981855 PMC8113732 33601913

[B24] Lou C. Xiao M. Cheng S. Lu X. Jia S. Ren Y. (2016). MiR-485-3p and miR-485-5p suppress breast cancer cell metastasis by inhibiting PGC-1α expression. Cell Death Dis. 7 (3), e2159. 10.1038/cddis.2016.27 27010860 PMC4823935

[B25] Lu T. X. Rothenberg M. E. (2018). MicroRNA. J. Allergy Clin. Immunol. 141 (4), 1202–1207. 10.1016/j.jaci.2017.08.034 29074454 PMC5889965

[B26] Lu Z. Gao Y. (2021). Screening differentially expressed genes between endometriosis and ovarian cancer to find new biomarkers for endometriosis. Ann. Med. 53 (1), 1377–1389. 10.1080/07853890.2021.1966087 34409913 PMC8381947

[B27] Lv X. Chen P. Liu W. (2015). Down regulation of MiR-93 contributes to endometriosis through targeting MMP3 and VEGFA. Am. J. Cancer Res. 5 (5), 1706–1717. 26175939 PMC4497437

[B28] Ma Z. Wang L. Z. Cheng J. T. Lam W. S. T. Ma X. Xiang X. (2021). Targeting hypoxia-inducible factor-1-mediated metastasis for cancer therapy. Antioxid. Redox Signal. 34 (18), 1484–1497. 10.1089/ars.2019.7935 33198508

[B29] Maier I. M. Maier A. C. (2021). miRNAs and lncRNAs: Potential non-invasive biomarkers for endometriosis. Biomedicines 9 (11), 1662. 10.3390/biomedicines9111662 34829891 PMC8615815

[B30] Malandrino A. Mak M. Kamm R. D. Moeendarbary E. (2018). Complex mechanics of the heterogeneous extracellular matrix in cancer. Extreme Mech. Lett. 21, 25–34. 10.1016/j.eml.2018.02.003 30135864 PMC6097546

[B31] Mohamed A. A. Omar A. A. A. El-Awady R. R. Hassan S. M. A. Eitah W. M. S. Ahmed R. (2020). MiR-155 and MiR-665 role as potential non-invasive biomarkers for hepatocellular carcinoma in Egyptian patients with chronic hepatitis C virus infection. J. Transl. Int. Med. 8 (1), 32–40. 10.2478/jtim-2020-0006 32435610 PMC7227164

[B32] Nasu K. Aoyagi Y. Zhu R. Okamoto M. Kai K. Kawano Y. (2022). Promising therapeutic targets of endometriosis obtained from microRNA studies. Med. Mol. Morphol. 55 (2), 85–90. 10.1007/s00795-021-00308-3 34846581

[B33] Nikoo S. Ebtekar M. Jeddi-Tehrani M. Shervin A. Bozorgmehr M. Vafaei S. (2014). Menstrual blood-derived stromal stem cells from women with and without endometriosis reveal different phenotypic and functional characteristics. Mol. Hum. Reprod. 20 (9), 905–918. 10.1093/molehr/gau044 24939730

[B34] Nisenblat V. Bossuyt P. M. Shaikh R. Farquhar C. Jordan V. Scheffers C. S. (2016). Blood biomarkers for the non-invasive diagnosis of endometriosis. Cochrane Database Syst. Rev. 2016 (5), Cd012179. 10.1002/14651858.CD012179 PMC7076288 27132058

[B35] Oana S. M. Claudia B. Lelia R. A. Simona M. Claudia C. Daniela D. E. (2022). Differential expression of tissular miRNA-155 in pediatric gastritis. J. Clin. Med. 11 (12), 3351. 10.3390/jcm11123351 35743416 PMC9224896

[B36] Okoth K. Wang J. Zemedikun D. Thomas G. N. Nirantharakumar K. Adderley N. J. (2021). Risk of cardiovascular outcomes among women with endometriosis in the United Kingdom: A retrospective matched cohort study. Bjog 128 (10), 1598–1609. 10.1111/1471-0528.16692 33683770

[B37] Pope S. D. Medzhitov R. (2018). Emerging principles of gene expression programs and their regulation. Mol. Cell 71 (3), 389–397. 10.1016/j.molcel.2018.07.017 30075140

[B38] Proestling K. Birner P. Gamperl S. Nirtl N. Marton E. Yerlikaya G. (2015). Enhanced epithelial to mesenchymal transition (EMT) and upregulated MYC in ectopic lesions contribute independently to endometriosis. Reprod. Biol. Endocrinol. 13, 75. 10.1186/s12958-015-0063-7 26198055 PMC4511248

[B39] Pu F. Chen F. Zhang Z. Shi D. Zhong B. Lv X. (2022). Ferroptosis as a novel form of regulated cell death: Implications in the pathogenesis, oncometabolism and treatment of human cancer. Genes Dis. 9 (2), 347–357. 10.1016/j.gendis.2020.11.019 35224151 PMC8843993

[B40] Pugh C. W. Ratcliffe P. J. (2003). Regulation of angiogenesis by hypoxia: Role of the HIF system. Nat. Med. 9 (6), 677–684. 10.1038/nm0603-677 12778166

[B41] Rezk N. A. Lashin M. B. Sabbah N. A. (2021). MiRNA 34-a regulate SIRT-1 and Foxo-1 expression in endometriosis. Noncoding. RNA Res. 6 (1), 35–41. 10.1016/j.ncrna.2021.02.002 33718673 PMC7905260

[B42] Saliminejad K. Khorram Khorshid H. R. Soleymani Fard S. Ghaffari S. H. (2019). An overview of microRNAs: Biology, functions, therapeutics, and analysis methods. J. Cell. Physiol. 234 (5), 5451–5465. 10.1002/jcp.27486 30471116

[B43] Sanchez A. M. Vanni V. S. Bartiromo L. Papaleo E. Zilberberg E. Candiani M. (2017). Is the oocyte quality affected by endometriosis? A review of the literature. J. Ovarian Res. 10 (1), 43. 10.1186/s13048-017-0341-4 28701212 PMC5508680

[B44] Saunders P. T. K. Horne A. W. (2021). Endometriosis: Etiology, pathobiology, and therapeutic prospects. Cell 184 (11), 2807–2824. 10.1016/j.cell.2021.04.041 34048704

[B45] Shen L. Hong X. Liu Y. Zhou W. Zhang Y. (2020). The miR-25-3p/Sp1 pathway is dysregulated in ovarian endometriosis. J. Int. Med. Res. 48 (4), 300060520918437. 10.1177/0300060520918437 32299271 PMC7169359

[B46] Shen Y. Li X. Dong D. Zhang B. Xue Y. Shang P. (2018). Transferrin receptor 1 in cancer: A new sight for cancer therapy. Am. J. Cancer Res. 8 (6), 916–931. 30034931 PMC6048407

[B47] Taylor H. S. Kotlyar A. M. Flores V. A. (2021). Endometriosis is a chronic systemic disease: Clinical challenges and novel innovations. Lancet (London, Engl. 397 (10276), 839–852. 10.1016/S0140-6736(21)00389-5 33640070

[B48] Tian F. J. An L. N. Wang G. K. Zhu J. Q. Li Q. Zhang Y. Y. (2014). Elevated microRNA-155 promotes foam cell formation by targeting HBP1 in atherogenesis. Cardiovasc. Res. 103 (1), 100–110. 10.1093/cvr/cvu070 24675724

[B49] Uchida T. Rossignol F. Matthay M. A. Mounier R. Couette S. Clottes E. (2004). Prolonged hypoxia differentially regulates hypoxia-inducible factor (HIF)-1alpha and HIF-2alpha expression in lung epithelial cells: Implication of natural antisense HIF-1alpha. J. Biol. Chem. 279 (15), 14871–14878. 10.1074/jbc.M400461200 14744852

[B50] Utsunomiya H. Cheng Y. H. Lin Z. Reierstad S. Yin P. Attar E. (2008). Upstream stimulatory factor-2 regulates steroidogenic factor-1 expression in endometriosis. Mol. Endocrinol. 22 (4), 904–914. 10.1210/me.2006-0302 18165439 PMC2276471

[B51] Vanhie A. Peterse D. Beckers A. Cuéllar A. Fassbender A. Hooghe T. D. (2019). Plasma miRNAs as biomarkers for endometriosis. Hum. Reprod. 34 (9), 1650–1660. 10.1093/humrep/dez116 31411334 PMC6736379

[B52] Vercellini P. Viganò P. Somigliana E. Fedele L. (2014). Endometriosis: Pathogenesis and treatment. Nat. Rev. Endocrinol. 10 (5), 261–275. 10.1038/nrendo.2013.255 24366116

[B53] Vishnoi A. Rani S. (2017). MiRNA biogenesis and regulation of diseases: An overview. Methods Mol. Biol. 1509, 1–10. 10.1007/978-1-4939-6524-3_1 27826912

[B54] Wattanayingcharoenchai R. Rattanasiri S. Charakorn C. Attia J. Thakkinstian A. (2021). Postoperative hormonal treatment for prevention of endometrioma recurrence after ovarian cystectomy: A systematic review and network meta-analysis. Bjog 128 (1), 25–35. 10.1111/1471-0528.16366 32558987 PMC7754428

[B55] Wong T. L. Che N. Ma S. (2017). Reprogramming of central carbon metabolism in cancer stem cells. Biochim. Biophys. Acta. Mol. Basis Dis. 1863 (7), 1728–1738. 10.1016/j.bbadis.2017.05.012 28502706

[B56] Xiao X. Gu Y. Wang G. Chen S. (2019). c-Myc, RMRP, and miR-34a-5p form a positive-feedback loop to regulate cell proliferation and apoptosis in multiple myeloma. Int. J. Biol. Macromol. 122, 526–537. 10.1016/j.ijbiomac.2018.10.207 30389523

[B57] Yu C. Li Y. Chen H. Yang S. Xie G. (2008). Decreased expression of aromatase in the Ishikawa and RL95-2 cells by the isoflavone, puerarin, is associated with inhibition of c-jun expression and AP-1 activity. Food Chem. Toxicol. 46 (12), 3671–3676. 10.1016/j.fct.2008.09.045 18848966

[B58] Yu G. Wang L. G. Han Y. He Q. Y. (2012). clusterProfiler: an R package for comparing biological themes among gene clusters. Omics 16 (5), 284–287. 10.1089/omi.2011.0118 22455463 PMC3339379

[B59] Zhang H. M. Kuang S. Xiong X. Gao T. Liu C. Guo A. Y. (2015). Transcription factor and microRNA co-regulatory loops: Important regulatory motifs in biological processes and diseases. Brief. Bioinform. 16 (1), 45–58. 10.1093/bib/bbt085 24307685

[B60] Zhang S. He Y. Liu C. Li G. Lu S. Jing Q. (2020). miR-93-5p enhances migration and invasion by targeting RGMB in squamous cell carcinoma of the head and neck. J. Cancer 11 (13), 3871–3881. 10.7150/jca.43854 32328191 PMC7171485

[B61] Zhao L. Gu C. Ye M. Zhang Z. Li L. Fan W. (2018). Integration analysis of microRNA and mRNA paired expression profiling identifies deregulated microRNA-transcription factor-gene regulatory networks in ovarian endometriosis. Reprod. Biol. Endocrinol. 16 (1), 4. 10.1186/s12958-017-0319-5 29357938 PMC5776778

[B62] Zheng J. Dai Y. Lin X. Huang Q. Shi L. Jin X. (2021). Hypoxia-induced lactate dehydrogenase A protects cells from apoptosis in endometriosis. Mol. Med. Rep. 24 (3), 637. 10.3892/mmr.2021.12276 34278456 PMC8281285

[B63] Zhou J. Ding Z. M. Hardiman P. J. (2018). Understanding the role of gui-zhi-fu-ling-capsules (Chinese medicine) for treatment of endometriosis in the rat model: Using NMR based metabolomics. Evid. Based. Complement. Altern. Med. 2018, 9864963. 10.1155/2018/9864963 PMC6313965 30662514

